# Supplementary material for: DEMIST: A Deep-Learning-Based Detection-Task-Specific Denoising Approach for Myocardial Perfusion SPECT
Source: IEEE Trans Radiat Plasma Med Sci. Author manuscript; Available in PMC 2024 May 17. (PMC11101197; doi:10.1109/trpms.2024.3379215)
Supplement: supp1-3379215 [file NIHMS1982748-supplement-supp1-3379215.pdf]

Supplementary Material

DEMIST: A deep-learning-based task-specific denoising approach for myocardial perfusion SPECT

Md Ashequr Rahman, Zitong Yu, Richard Laforest, Craig K. Abbey, Barry A. Siegel, and Abhinav K. Jha*

M. A. Rahman and Z. Yu are with the Department of Biomedical Engineering, Washington University, St. Louis, MO 63130 USA.

C. K. Abbey is with the Department of Psychological and Brain Sciences, University of California, Santa Barbara, CA 93106 USA.

R. Laforest and B. A. Siegel are with the Mallinckrodt Institute of Radiology, Washington University, St. Louis, MO 63130 USA.

*A. K. Jha is with the Department of Biomedical Engineering and Mallinckrodt Institute of Radiology, Washington University, St. Louis, MO 63130 USA (e-mail: a.jha@wustl.edu).

S-I. Network architecture

TABLE S-1
Network architecture (Conv. = convolutional, BN = Batch Normalization, ReLU = Rectified Linear Unit, MaxPool = Max Pooling).

Layer	Layer type	Number of filters	Filter size	Stride/Pool size	Input size	Output size
1	Conv.	16	3×3×3	1×1×1	48×48×48×1	48×48×48×16
2	BN + Leaky ReLU	-	-	-	48×48×48×16	48×48×48×16
3	MaxPool	-	-	2×2×2	24×24×24×16	24×24×24×16
4	Conv.	32	3×3×3	1×1×1	24×24×24×16	24×24×24×32
5	BN + Leaky ReLU	-	-	-	24×24×24×32	24×24×24×32
6	MaxPool	-	-	2×2×2	12×12×12×32	12×12×12×32
7	Conv.	64	3×3×3	1×1×1	12×12×12×32	12×12×12×64
8	BN + Leaky ReLU	-	-	-	12×12×12×64	12×12×12×64
9	MaxPool	-	-	2×2×2	12×12×12×64	6×6×6×64
10	Conv.	128	3×3×3	1×1×1	6×6×6×64	6×6×6×128
11	BN + Leaky ReLU	-	-	-	6×6×6×128	6×6×6×128
12	Dropout	-	-	-	6×6×6×128	6×6×6×128
13	Transposed Conv.	64	3×3×3	2×2×2	6×6×6×128	12×12×12×64
14	BN + Leaky ReLU	-	-	-	12×12×12×64	12×12×12×64
15	Add Layer 8	-	-	-	12×12×12×64	12×12×12×64
16	Conv.	64	3×3×3	1×1×1	12×12×12×64	12×12×12×64
17	BN + Leaky ReLU	-	-	-	12×12×12×64	12×12×12×64
18	Transposed Conv.	32	3×3×3	2×2×2	12×12×12×64	24×24×24×32
19	BN + Leaky ReLU	-	-	-	24×24×24×32	24×24×24×32
20	Add Layer 5	-	-	-	24×24×24×32	24×24×24×32
21	Conv.	32	3×3×3	1×1×1	24×24×24×32	24×24×24×32
22	BN + Leaky ReLU	-	-	-	24×24×24×32	24×24×24×32
23	Transposed Conv.	16	3×3×3	2×2×2	24×24×24×32	48×48×48×16
24	BN + Leaky ReLU	-	-	-	48×48×48×16	48×48×48×16
25	Add Layer 2	-	-	-	48×48×48×16	48×48×48×16
26	Conv.	16	3×3×3	1×1×1	48×48×48×16	48×48×48×16
27	BN + Leaky ReLU	-	-	-	48×48×48×16	48×48×48×16
28	Conv	1	1×1×1	1×1×1	48×48×48×1	48×48×48×1
29	BN + Leaky ReLU	-	-	-	48×48×48×1	48×48×48×1

S-II. Evaluation with channelized multi-template observer

A. Background

To assess the robustness of DEMIST method across various channelized model observers, we performed our objective evaluation study using a different channelized model observer, namely a channelized multi-template observer (CMTO). In our population, the defect sizes, severities and locations were all varying. In this case, it has been observed that the channel outputs (vectors) for the entire population may not be multivariate normally distributed [1], thus limiting the applicability of the widely known Hotelling observer. However, the channel outputs for sub-ensembles of patient data grouped based on defect type may have multivariate normal distributions [2]. For this case, a CMTO was developed and evaluated in the context of MPI SPECT [2]. The CMTO applies the Hotelling template to the channel outputs and adds a constant term to compute test statistics for each sub-ensemble, and calculates a single global area under the ROC curve (AUC) using the pooled test statistics from all the sub-ensembles. The observer yields the maximal AUC when shifting the distributions of Hotelling observer test statistics by a different constant for each sub-ensemble is allowed [2]. The channels chosen for this observer were also the rotationally symmetric frequency channels, as in the CHO study. Also, the clinical task for this observer was detecting perfusion defects where the defect location was known.

B. Generation of test statistic

We followed the same procedure as described in Sec. III-C of the manuscript to obtain channel vectors. For each sub-ensemble, the channel vectors of defect-present and defect-absent population were used to learn the template using a leave-one-out approach. The collection of test statistic from each sub-ensemble were then pooled [2] and were used to perform the ROC analysis.

C. Results

Fig. S-1 shows the AUC values obtained with the low-dose protocol, TADL method, DEMIST method and normal-dose protocol. We observed that at all dose levels, the DEMIST method yielded a significant improvement ($p < 0.05$) in performance on detection task compared to the low-dose protocol as well as the TADL method. The TADL method generally did not improve performance compared to the low-dose protocol.

Figs. S-2 and S-3 show the AUC values obtained for stratified analysis based on sex. We observed that at all dose levels and stratified groups, the DEMIST method yielded a significant improvement ($p < 0.05$) in performance on detection task compared to the low-dose protocol and TADL method. Again, we observed that the TADL method generally did not improve performance significantly compared to the low-dose protocol.

Figs. S-4 and S-5 show the AUC values obtained for stratified analysis based on defect extent and severity, respectively. Similar to previous results, we observed that at all dose levels and stratified groups, the DEMIST method yielded a significant improvement ($p < 0.05$) in performance on detection task compared to low-dose protocol and TADL method. Again, the TADL method generally did not improve performance significantly compared to the low-dose protocol.

Fig. S-6 shows the AUC values obtained for stratified analysis based on scanner type. We observed that at all dose levels, for both NaI and CZT scanners, the DEMIST method yielded a significant improvement ($p < 0.05$) in performance on detection task compared to low-dose protocol and TADL method. The TADL method generally did not improve performance significantly compared to the low-dose protocol.

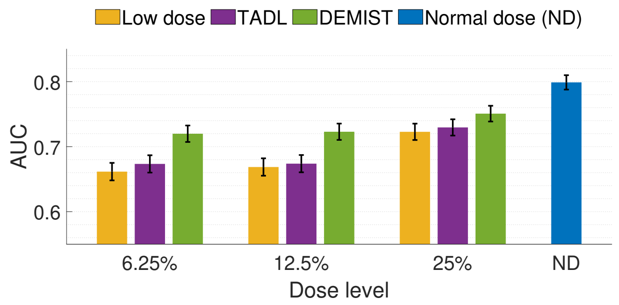

Fig. S-1. AUC values obtained for the normal and low-dose images, the images denoised using the proposed DEMIST approach and TADL approach at various dose levels with CMTO. Error bars denote 95% confidence intervals.

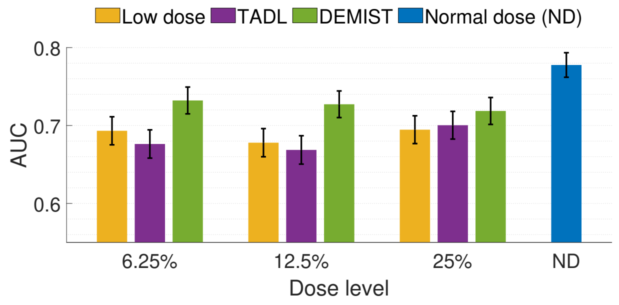

Fig. S-2. AUC values for male patients obtained for the different approaches and at various dose levels using CMTO. Error bars denote 95% confidence intervals.

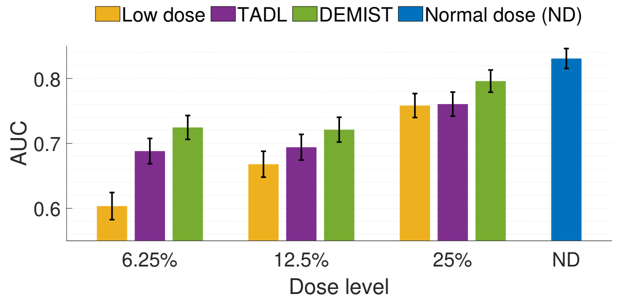

Fig. S-3. AUC values obtained for the different approaches and at various dose levels with female patients using CMTO. Error bars denote 95% confidence intervals.

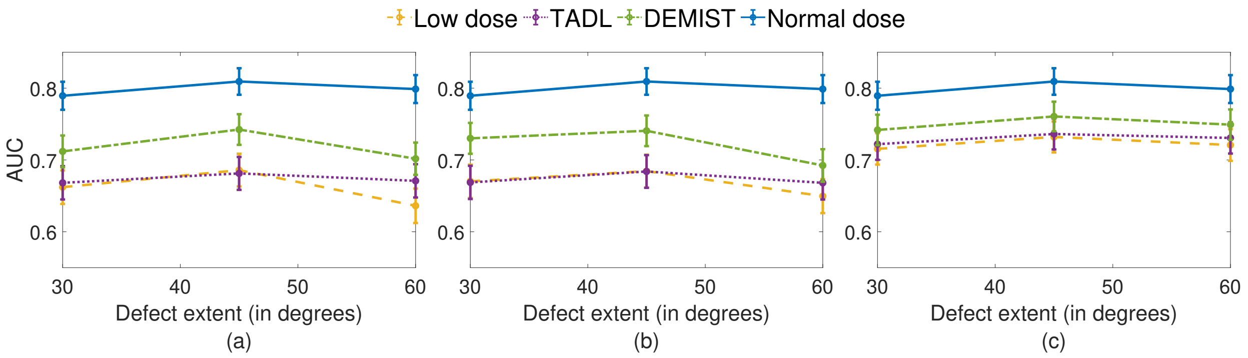

Fig. S-4. AUC values obtained using CMTO for the various approaches as a function of different defect extents with (a) 6.25%, (b) 12.5% and (c) 25% dose levels. Error bars denote 95% confidence intervals.

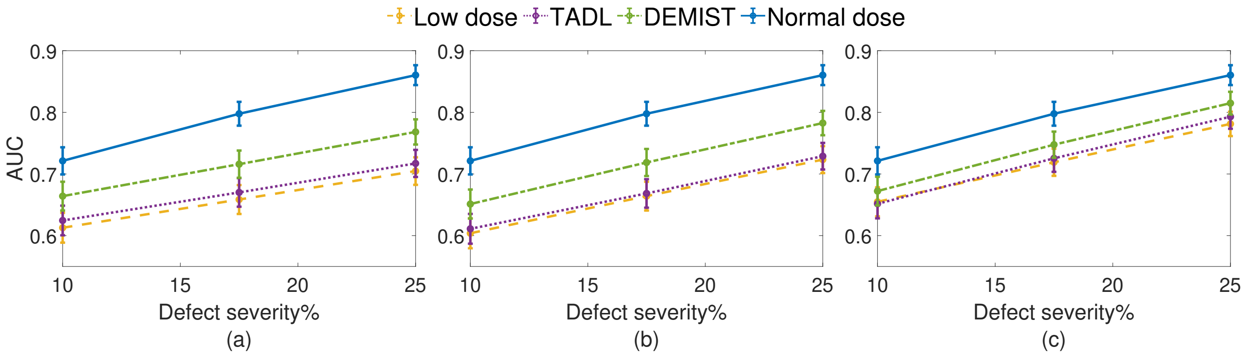

Fig. S-5. AUC values obtained using CMTO for the various approaches as a function of different defect severities with (a) 6.25%, (b) 12.5% and (c) 25% dose levels. Error bars denote 95% confidence intervals.

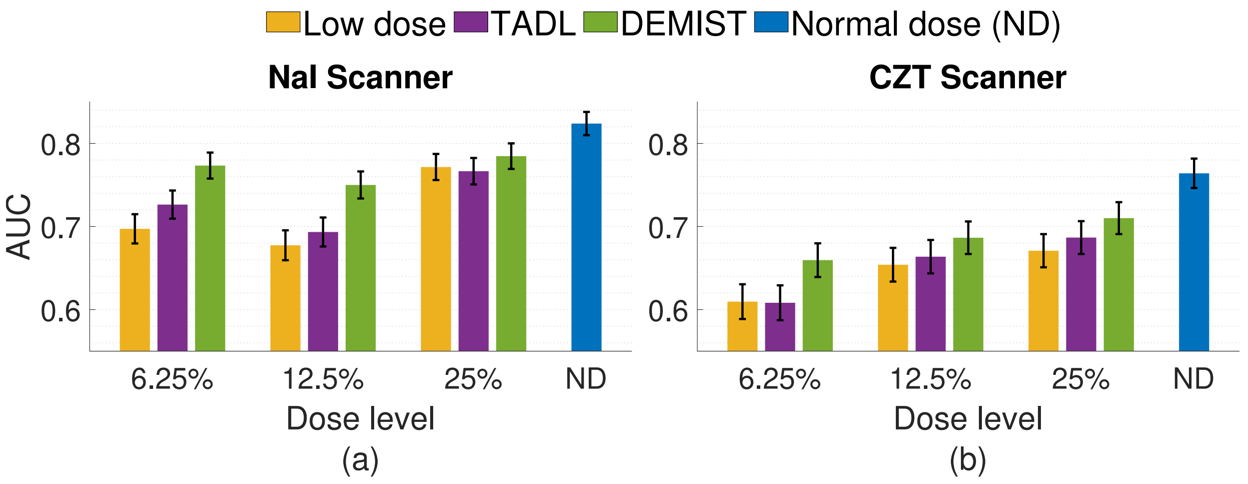

Fig. S-6. AUC values obtained using CMTO for the various approaches for (a) NaI and (b) CZT scanner at various dose levels. Error bars denote 95% confidence intervals.

S-III. Corrected p-values of DeLong's test for AUC comparison between two methods

TABLE S-2
Corrected p-values for various analyses and observers (LD = low-dose protocol)

Analysis type	Stratified group	Observer	Comparisons	Dose levels		
				6.25%	12.50%	25%
Non-stratified analysis	-	CMTO	DEMIST VS LD	2.72E-32	8.05E-38	1.44E-28
			DEMIST VS TADL	2.74E-29	1.19E-29	2.08E-14
			TADL VS LD	0.111834	0.840879	0.079587
		CHO	DEMIST VS LD	8.78E-20	5.72E-10	2.64E-17
			DEMIST VS TADL	5.87E-11	3.71E-17	6.22E-08
			TADL VS LD	0.014445	1.634715	0.035604
Stratified analysis based on sex	Male	CMTO	DEMIST VS LD	4.43E-07	1.83E-15	4.8E-09
			DEMIST VS TADL	2.33E-18	4.39E-18	0.000553
			TADL VS LD	0.130437	0.626301	0.607878
		CHO	DEMIST VS LD	0.002403	4.71E-05	7.44E-07
			DEMIST VS TADL	8.25E-10	0.000263	0.018342
			TADL VS LD	0.130599	1.255887	0.350955
	Female	CMTO	DEMIST VS LD	2.59E-49	1.21E-16	1.13E-26
			DEMIST VS TADL	5.46E-05	1.04E-05	3.56E-17
			TADL VS LD	6.51E-15	0.001287	1.792665
		CHO	DEMIST VS LD	1.51E-18	3.47E-07	2.46E-13
			DEMIST VS TADL	0.132264	1.27E-17	1.94E-08
			TADL VS LD	8.02E-11	0.200286	0.41967
Stratified analysis based on defect extent	30° extent	CMTO	DEMIST VS LD	2.08E-08	4.02E-15	1.04E-09
			DEMIST VS TADL	7.07E-10	5.29E-14	4.73E-05
			TADL VS LD	1.581237	2.56014	0.606618
		CHO	DEMIST VS LD	1.4E-05	0.025992	1.57E-06
			DEMIST VS TADL	2.42E-05	0.000472	0.00423
			TADL VS LD	1.077156	2.296377	0.362862
	45° extent	CMTO	DEMIST VS LD	1.1E-11	1.08E-15	9.54E-13
			DEMIST VS TADL	1.37E-17	4.72E-15	1.53E-06
			TADL VS LD	1.904022	2.836206	1.246464
		CHO	DEMIST VS LD	2.75E-09	2.93E-05	0.000266
			DEMIST VS TADL	5.04E-07	4.47E-08	0.004428
			TADL VS LD	0.681075	1.96272	2.055168
	60° extent	CMTO	DEMIST VS LD	1.31E-13	4.45E-08	4.55E-08
			DEMIST VS TADL	7.56E-05	0.002286	0.000327
			TADL VS LD	0.002466	0.130788	0.251469
		CHO	DEMIST VS LD	1.08E-10	2.12E-06	2.58E-08
			DEMIST VS TADL	0.088839	3.15E-07	0.001602
			TADL VS LD	0.000753	1.500372	0.080946
Stratified analysis based on defect severity	10% severity	CMTO	DEMIST VS LD	1.11E-07	1.21E-07	0.001269
			DEMIST VS TADL	4.07E-07	6.94E-06	0.000468
			TADL VS LD	0.784584	1.289538	1.785861
		CHO	DEMIST VS LD	0.000543	0.038628	0.001143
			DEMIST VS TADL	0.142011	0.021213	0.055386
			TADL VS LD	0.346419	2.395917	0.808731
	17.5% severity	CMTO	DEMIST VS LD	2.88E-11	1.56E-13	2.73E-10
			DEMIST VS TADL	2.83E-10	5.92E-11	9.09E-06
			TADL VS LD	0.719325	1.793304	0.692793
		CHO	DEMIST VS LD	2.35E-07	0.000505	9.99E-07
			DEMIST VS TADL	0.00026	1.07E-06	0.001629
			TADL VS LD	0.312813	2.061603	0.514611
	25% severity	CMTO	DEMIST VS LD	7.94E-16	5.31E-21	1.33E-17
			DEMIST VS TADL	3.04E-14	1.6E-15	3.68E-07
			TADL VS LD	0.542664	1.39266	0.038385
		CHO	DEMIST VS LD	1.58E-12	4.38E-07	6.98E-10
			DEMIST VS TADL	1.13E-09	3.32E-13	6.31E-05
			TADL VS LD	0.260334	1.118241	0.340011

TABLE S-3
CORRECTED P-VALUES FOR STRATIFIED ANALYSIS WITH SCANNER TYPES

Analysis type	Stratified group	Observer	Comparisons	Dose levels		
				6.25%	12.50%	25%
Stratified analysis based on scanner	NaI	CMTO	DEMIST VS LD	1.79E-38	4.33E-37	3.59E-06
			DEMIST VS TADL	1.50E-21	2.24E-21	4.51E-07
			TADL VS LD	8.97E-05	0.05029	0.428633
		CHO	DEMIST VS LD	1.79E-14	1.59E-08	2.554406
			DEMIST VS TADL	0.00164	0.454727	0.057514
			TADL VS LD	4.93E-06	0.000214	0.066835
	CZT	CMTO	DEMIST VS LD	6.16E-08	4.68E-06	4.50E-13
			DEMIST VS TADL	1.48E-08	0.00161	3.14E-05
			TADL VS LD	2.692152	0.64725	0.018067
		CHO	DEMIST VS LD	0.00263	1.830386	1.88E-16
			DEMIST VS TADL	0.000259	5.87E-11	4.20E-05
			TADL VS LD	2.378452	6.44E-06	0.000155

S-IV. SPECT SCANNER CONFIGURATION

TABLE S-4
ACQUISITION AND RECONSTRUCTION PARAMETERS OF SPECT/CT SYSTEMS. (LEHR=LOW-ENERGY HIGH-RESOLUTION,WEHR=WIDE-ENERGY HIGH-RESOLUTION)

	Scanner	
	GE Discovery NM/CT 670 Pro NaI	GE Discovery NM/CT 670 Pro CZT
Number of cases in test data	63	51
Number of cases in train data	102	82
Number of cases in validation data	12	28
Collimator type	LEHR	WEHR
Collimator grid	Parallel hole	Parallel hole
Detector	NaI	CZT
Energy resolution at 140 keV (%)	9.8	6.3
Intrinsic spatial resolution (in mm)	3.9	2.46
System sensitivity (cps/MBq) at 10 cm	72	85
Photopeak energy window (in keV)	126-154	126-154
Reconstruction	OSEM	OSEM
Subsets	6	6
Iteration	8	8
Attenuation correction	CT	CT

S-V. EXAMPLES OF DEFECT SIGNALS

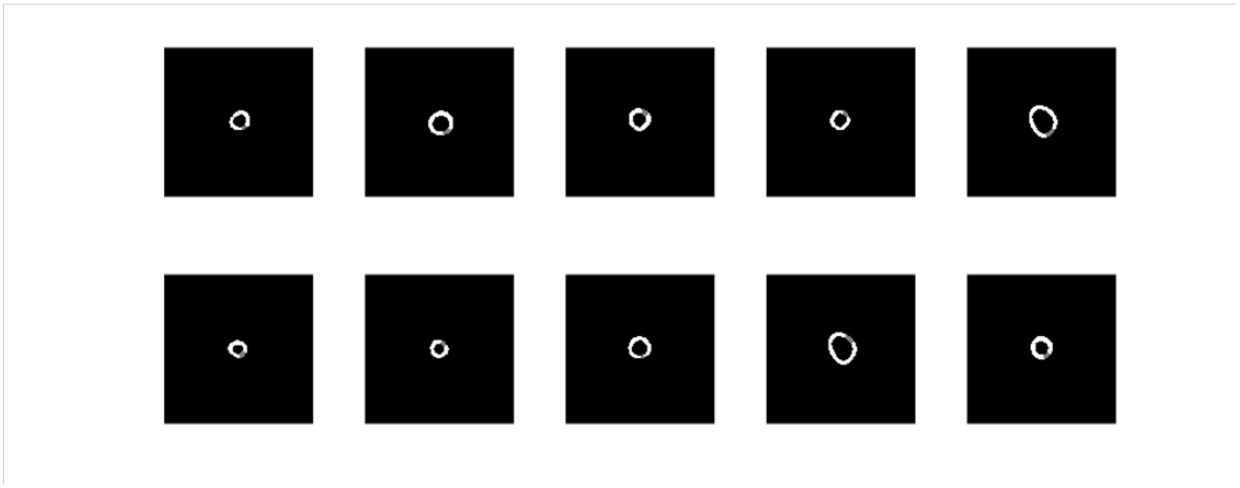

Fig. S-7. Examples of inserted defect using the LV segmented mask with varying extents and locations. The defects are at 50% severity for illustration purpose.

S-VI. ROC CURVES OF NON-STRATIFIED AND STRATIFIED ANALYSIS

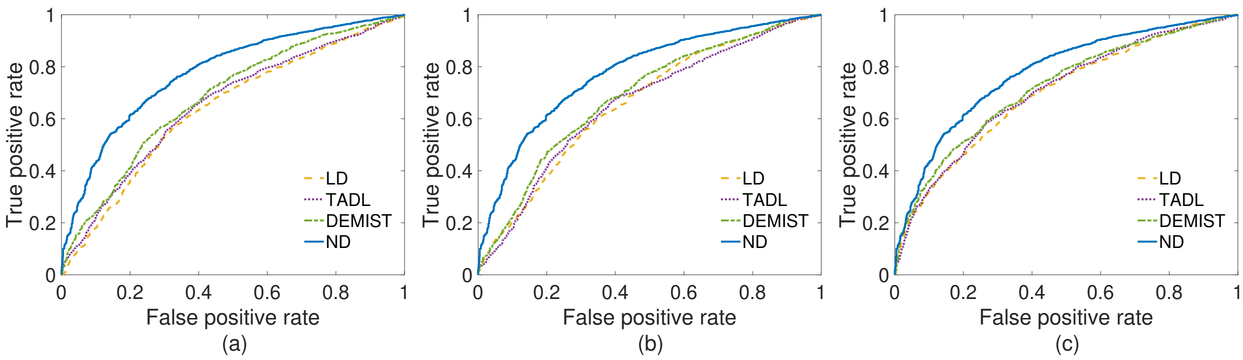

Fig. S-8. ROC curves for different approaches with (a) 6.25%, (b) 12.5% and (c) 25% dose levels with CHO.

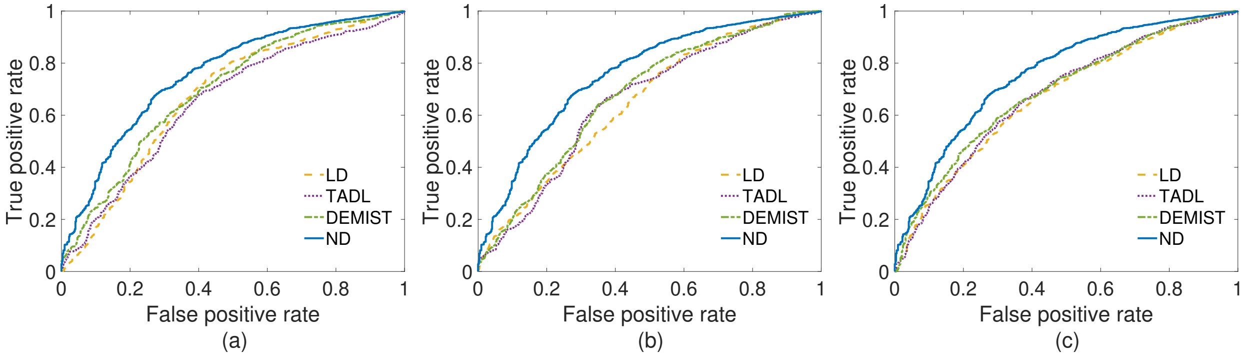

Fig. S-9. ROC curves for male patients with different approaches at (a) 6.25%, (b) 12.5% and (c) 25% dose levels with CHO.

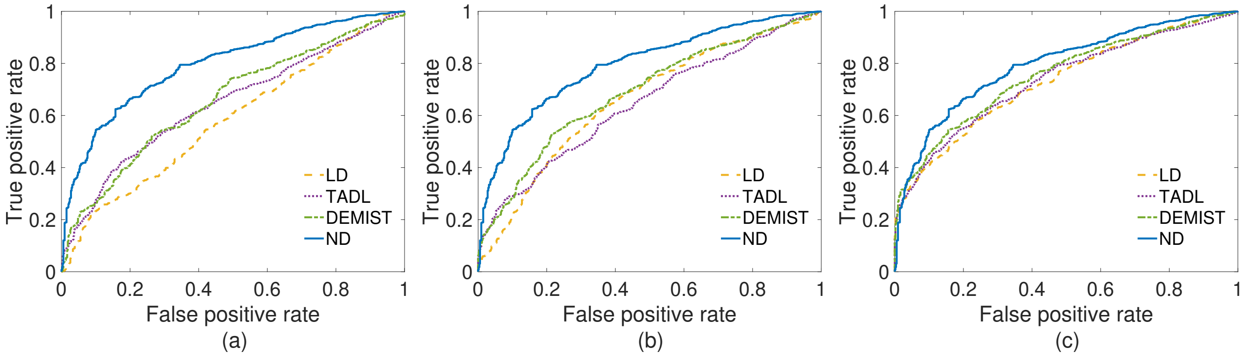

Fig. S-10. ROC curves for female patients with different approaches at (a) 6.25%, (b) 12.5% and (c) 25% dose levels with CHO.

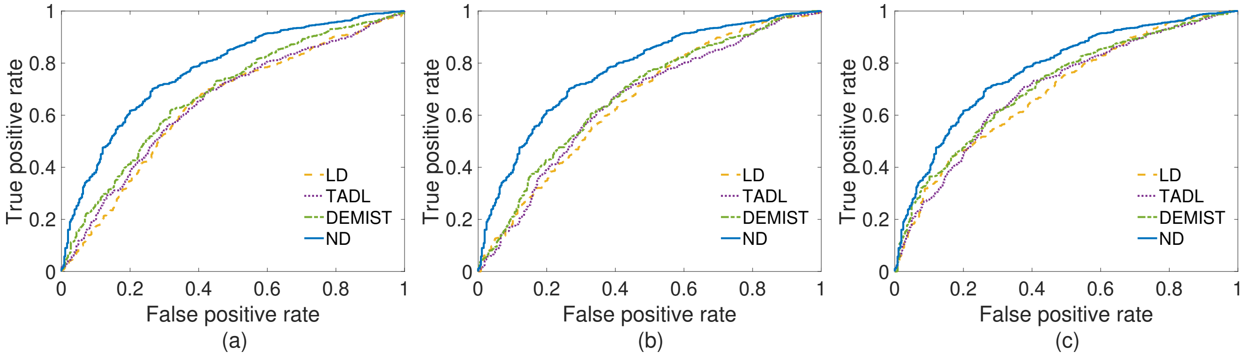

Fig. S-11. ROC curves for 30° defect extent with different approaches at (a) 6.25%, (b) 12.5% and (c) 25% dose levels with CHO.

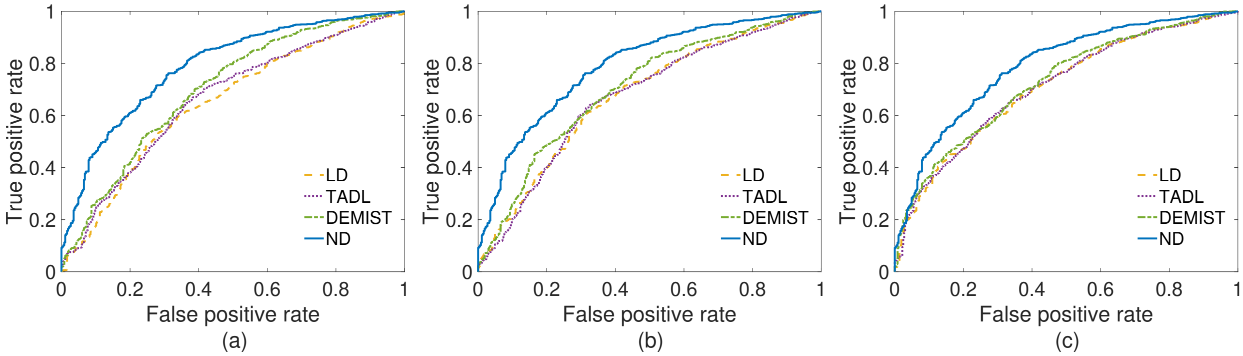

Fig. S-12. ROC curves for 45° defect extent with different approaches at (a) 6.25%, (b) 12.5% and (c) 25% dose levels with CHO.

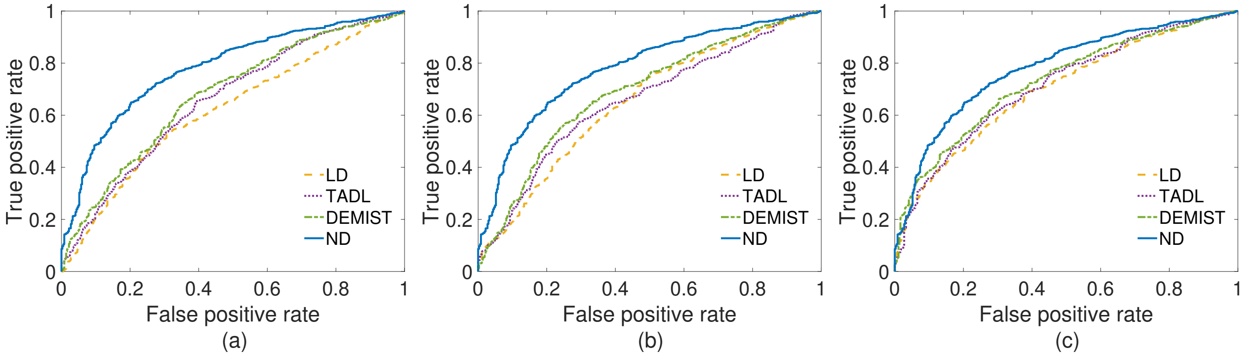

Fig. S-13. ROC curves for 60° defect extent with different approaches at (a) 6.25%, (b) 12.5% and (c) 25% dose levels with CHO.

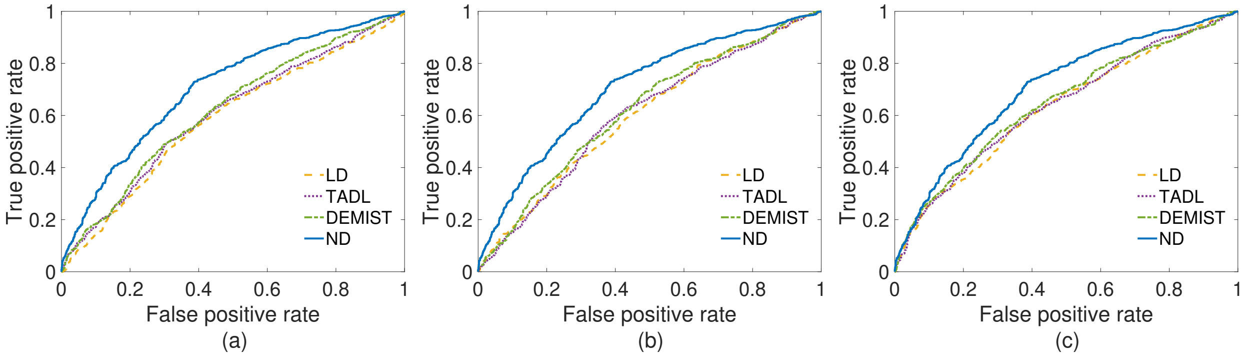

Fig. S-14. ROC curves for 10% defect severity with different approaches at (a) 6.25%, (b) 12.5% and (c) 25% dose levels with CHO.

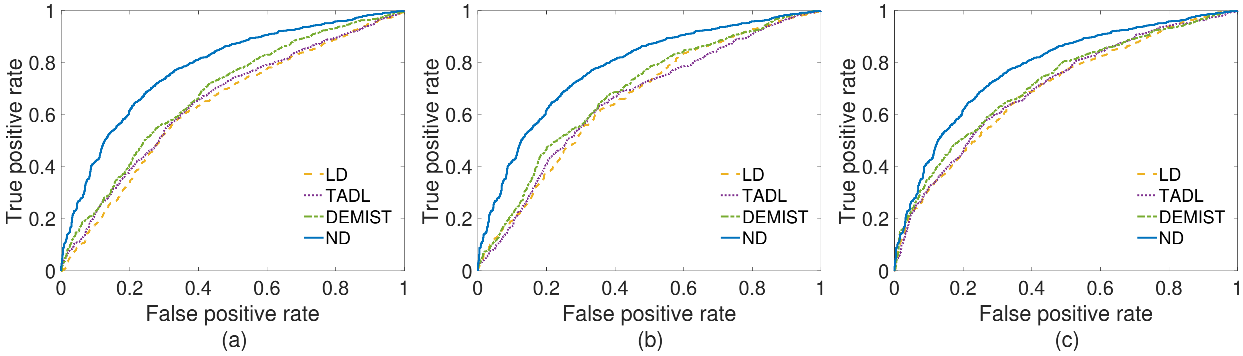

Fig. S-15. ROC curves for 17.5% defect severity with different approaches at (a) 6.25%, (b) 12.5% and (c) 25% dose levels with CHO.

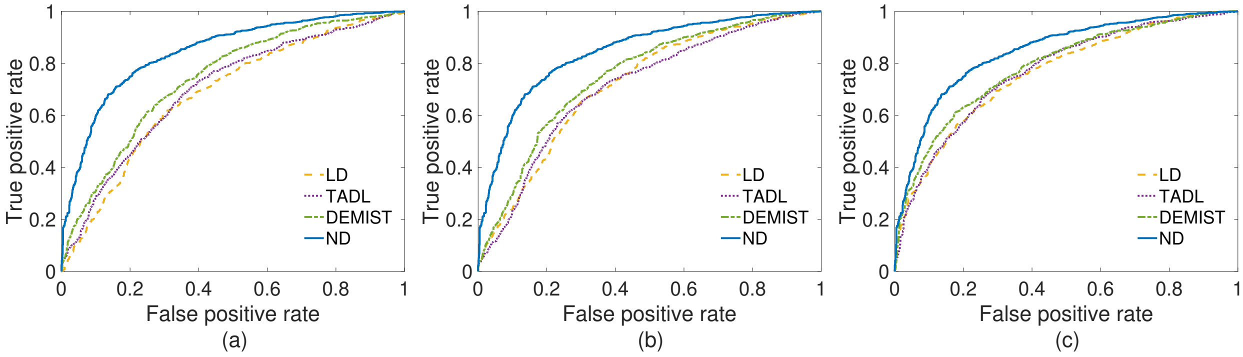

Fig. S-16. ROC curves for 25% defect severity with different approaches at (a) 6.25%, (b) 12.5% and (c) 25% dose levels with CHO.

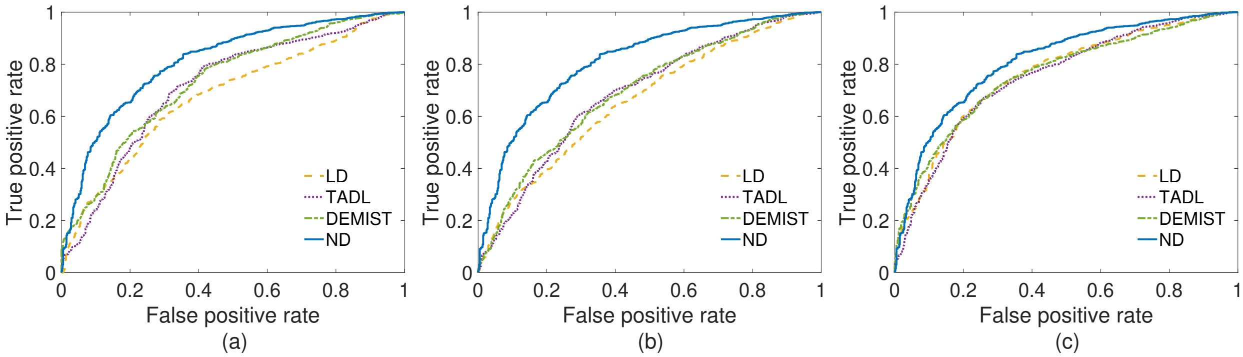

Fig. S-17. ROC curves for NaI scanner with different approaches at (a) 6.25%, (b) 12.5% and (c) 25% dose levels with CHO.

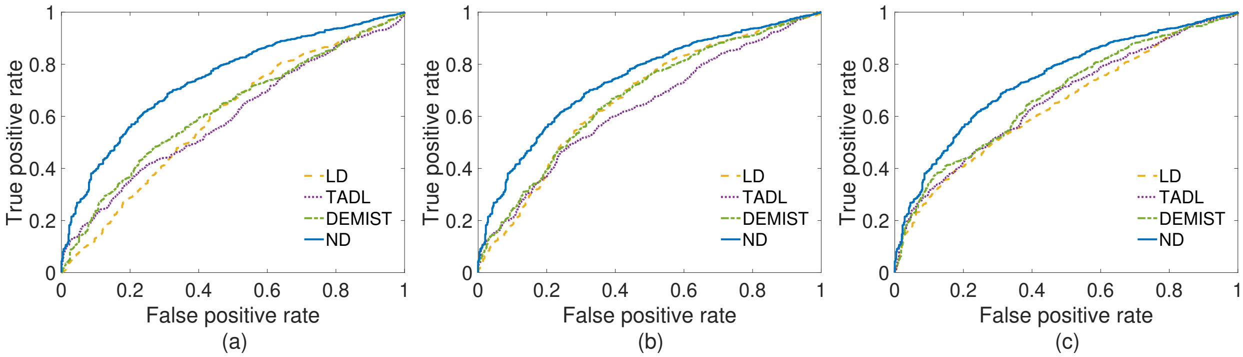

Fig. S-18. ROC curves for CZT scanner with different approaches at (a) 6.25%, (b) 12.5% and (c) 25% dose levels with CHO.

S-VII. Eigenvalues of noise covariance matrix

TABLE S-5
Eigenvalues of noise covariance matrix for defect type 1 and 2 in Fig. 10 of the manuscript

Defect type	eigenvalue index	DEMIST	TADL	Low dose	Normal dose
1	1	9.0339e+04	5.6555e+04	9.8293e+04	5.8214e+04
	2	895.7745	548.8621	1.7790e+03	565.8395
	3	16.2792	12.9378	54.3595	45.9154
	4	4.6574	9.1591	38.1404	12.2971
2	1	5.4074e+04	3.5064e+04	7.7168e+04	4.0850e+04
	2	650.7050	665.1538	2.0431e+03	593.1918
	3	37.1322	42.6869	42.1296	40.4913
	4	6.0972	7.1659	30.5935	23.7583

References

[1] F. E. Elshahaby, M. Ghaly, A. K. Jha, and E. C. Frey, "Factors affecting the normality of channel outputs of channelized model observers: an investigation using realistic myocardial perfusion SPECT images," *J. Med. Imaging*, vol. 3, no. 1, pp. 015 503–015 503, 2016.

[2] X. Li, A. K. Jha, M. Ghaly, F. E. Elshahaby, J. M. Links, and E. C. Frey, "Use of sub-ensembles and multi-template observers to evaluate detection task performance for data that are not multivariate normal," *IEEE Trans. Med. Imaging*, vol. 36, no. 4, pp. 917–929, 2016.
